# Can pre-existing medical conditions explain occupational differences in COVID-19 disease severity? An analysis of 3.17 million people insured in Germany

**DOI:** 10.5271/sjweh.4242

**Published:** 2025-09-01

**Authors:** Robert Guţu, Valerie Schaps, Benjamin Wachtler, Florian Beese, Jens Hoebel, Marco Alibone, Morten Wahrendorf

**Affiliations:** 1 Institute of Medical Sociology, Centre for Health and Society, Medical Faculty and University Hospital Düsseldorf, Heinrich-Heine University Düsseldorf, Düsseldorf, Germany.; 2 Division of Social Determinants of Health, Department of Epidemiology and Health Monitoring, Robert Koch Institute, Berlin, Germany.; 3 InGef - Institute for Applied Health Research Berlin GmbH, Berlin, Germany.

**Keywords:** vulnerable worker, susceptibility, pre-existing condition

## Abstract

**Objective:**

Occupational differences in COVID-19 are well documented, but the empirical evidence on potential reasons for these differences remains limited. Possible reasons include pre-existing health conditions. This study investigated occupational differences in COVID-19 disease severity and whether they can be attributed to pre-existing health conditions.

**Methods:**

Our study used German health insurance data covering 3.17 million insured individuals (age 18–67 years), with details on COVID-19-related hospitalization and mortality in 2020 and 2021, information on occupation (regrouped into four classifications) and pre-existing health conditions (divided into seven disease groups). In addition to descriptive statistics, we estimated multivariable Cox regression models with varying sets of adjustments.

**Results:**

We found clear occupational differences in COVID-19 hospitalization and mortality, with the highest risks for the production sector (especially manufacturing), commercial services (especially cleaning) and for low-skilled occupations. These findings persisted after adjusting for age, sex, and region, and also after mutual adjustment for other occupational classifications. We also found some evidence that the association between occupation and disease severity was partly explained by pre-existing conditions, especially in the case of low skill levels.

**Conclusions:**

Our findings provide support for occupational differences in COVID-19, where the occupational classifications under study were independently related to risk differences (eg, skill-level and job sector). Furthermore, we provide empirical evidence that differences by occupational skill levels are partly due to pre-existing conditions. This finding suggests that occupational inequalities in health increased during the pandemic, with those with poorer health who worked in disadvantaged occupations also being more likely to experience severe COVID-19 outcomes.

Numerous studies have shown that the risk of infection with SARS-CoV-2 and COVID-19 disease severity was unequally distributed among the population, with the socially disadvantaged population being at higher risk during the pandemic (1–4). Similar findings have been reported in a growing body of international studies investigating occupational differences in both infection risks and COVID-19 disease severity. Most of these studies rely on large-scale administrative datasets, including national health insurance records (eg, Germany, Denmark, Sweden) (5–12), population-based cohorts (eg, UK Biobank, Virus Watch) (13–15), national disease registries (9, 16, 17), or contact tracing and serological data (18). Sample sizes range from around 10 000 to more than 14 million individuals. Across countries and pandemic waves, studies consistently show that healthcare professionals, personal service workers, and employees in cleaning, transport, education, and food production faced elevated risks of infection and hospitalization. For example, analyses of German health insurance data indicated that not only medical personnel but also transport and logistics workers experienced increased hospitalization risks (10, 12). Similar patterns were observed in Sweden, where care workers and drivers were at elevated risk for both infection and severe disease (19), and in Denmark, where hospital admissions due to COVID-19 remained elevated for essential workers even in later waves (6). In the large-scale English study by Nafilyan et al (15), elevated COVID-19 mortality was observed in healthcare, social care, and transport sectors, even after adjusting for socioeconomic factors and medical history. The systematic review by Rhodes et al (20), which synthesizes evidence from 17 European studies, reinforces these patterns. It shows that while infection risks among healthcare workers declined over time due to improved protective measures, consistently elevated risks persisted across later pandemic waves in education, social care, and transport. Occupational risk was closely linked to workplace-specific factors such as frequent close contact with others, indoor working conditions, poor ventilation, and insufficient personal protective equipment—especially in the early phases of the pandemic. The review also highlights dynamic shifts over time: whereas healthcare and caregiving professions showed the highest infection risks early in the pandemic, manufacturing, logistics, and education became more affected in later stages, consistent with wave-specific findings from Germany (8). Several studies also report gender-specific patterns. For instance, some analyses found elevated infection or hospitalization risks in healthcare settings exclusively among women (10), while Torén et al (19) reported that the risk of severe COVID-19 was more pronounced for women than men in Swedish healthcare occupations (19). These findings may reflect both occupational segregation and differing task-related exposures within the same job categories.

While most studies assessed outcomes at the population level, few focused on the infected population too. This limits the ability to differentiate between infection risk and disease severity among those already infected—an important gap, given that vulnerability to infection and vulnerability to severe progression may follow distinct social and occupational patterns (12). Another shortcoming of previous studies is that measures of occupation were rather heterogeneous, often lacking a conceptual approach that accounts for both horizontal aspects of the job (eg, branches of industries) and vertical aspects (eg required skill levels). This again limits existing knowledge, specifically, far-reaching conclusions about which occupational aspects or dimensions – such as sector or skill – are crucial for the explanation of the reported differences (8, 10). This leads to another important limitation: While the descriptive evidence on occupational disparities described above is growing and convincing, we still know relatively little about the underlying mechanisms driving these well-documented differences.

At this point, inequalities in three main areas have been proposed as possible reasons for these differences: exposure, care and vulnerability (or “susceptibility”) (21–23). Inequalities in exposure means that the risk of being exposed to the virus varies between population groups and that disadvantaged populations and those working in particular occupations (eg, medical sector) are more likely to be exposed. For instance, disadvantaged occupational groups had fewer opportunities to reduce face-to-face contacts during the pandemic (24). Along these lines, for example, an ecological study from Germany compared levels of infections between regions and found that infections rates were higher in regions with higher employment rates, particularly in regions where the proportion of employment in the secondary sector was high (25). Conversely, the ability to work from home varies by jobs (26). Inequalities in care means that disadvantaged groups may have poorer access (eg, lower vaccination rates) and lower quality of medical care but also that population groups use medical care differently (ie, delayed symptoms awareness) or are less likely to profit from preventive measures (eg, less developed infection control measures for some occupations). In the German context, studies suggested that socioeconomically disadvantaged groups showed lower rates of being vaccinated or basic immunized against COVID-19 (2). The third explanation for inequalities – vulnerability – refers to higher prevalence of underlying health-conditions among socioeconomically disadvantaged groups, making them more “vulnerable” because of poorer health conditions (27). Medical preconditions are likely to be particularly relevant when examining disease severity, and – conceptually – may function as moderator, mediator, or confounder in the association between occupation and COVID-19. These roles are not mutually exclusive and may operate simultaneously. As a moderator, pre-conditions are expected to amplify the effect of occupation on COVID-19 risks, such that individuals in disadvantaged occupation with poor health are more severely affected than those with good health. As a mediator, poor health conditions, at least in part, would mediate the association between occupation and COVID-19 outcomes, where occupation cause differences in medical pre-condition that in turn increase the risk of COVID-19. If such pre-conditions existed before starting the job, however, these may also act as confounder. So far, surprisingly few studies investigated these possible mechanisms and the related empirical evidence remains scarce. In fact, various studies showed that pre-existing conditions have had an impact on disease severity (28–32), such as cardiovascular disease (29, 30, 32) or diabetes (30). However, only a few studies have comprehensively assessed the association between occupation and COVID-19, thereby, integrating information on pre-existing health conditions (6, 12, 15, 16).

Taken together, while numerous studies have documented occupational differences in COVID-19, the empirical evidence on potential explanations is still limited. This includes a lack of studies focusing specifically on infected populations (when studying disease severity) and the absence of conceptually grounded occupational measures that distinguish between vertical and horizontal dimension of the job. Using health insurance data for over three million men and women aged 18–67 years, this study aimed to address these shortcomings and to explore the potential mediation role of pre-existing health conditions in the association between occupation and COVID-19 outcomes.

## Methods

### Data sources

We used data from the Institute for Applied Health Research's (InGef) Research Database (RDB) (33), which contains anonymized longitudinal claims data on about 6.8 million insured persons. These were provided to InGef by about 60 statutory health insurances throughout Germany. The data consisted of basic socio-demographic information on the insured, International Statistical Classification of Diseases and Related Health Problems Version 10 (ICD-10) diagnoses, information on outpatient and inpatient treatments and procedures, as well as details on drug prescriptions and incapacity to work. In addition, there are employer-provided occupation details [based on the occupation code (TTS), see below]. The data were anonymized and did not allow for any conclusions to be drawn about individual insured persons, individual service providers (ie, doctors, practices, hospitals, pharmacies) or the portfolio of individual health insurance funds. InGef exclusively carried out the analyses in a protected environment in accordance with data protection guidelines (Federal Data Protection Act) and the recommendations of the Good Epidemiological Practice of the German Society for Epidemiology (34).

### Study population

The initial study sample consisted of 6.8 million men and women who were part of InGef's RDB on 1 January 2020. Of these, only individuals of working age (18–67 years at the beginning of the observation period) were included. Additionally, only those for whom information was available in the RDB for the year 2019, such as data on pre-existing medical conditions, were considered. Finally, insured persons with missing information on occupation were excluded. This resulted in a net sample of 3 173 171 insured persons (approximately 47% women), which corresponds to about 10% of all employees subject to social insurance contributions in Germany in January 2020 (about 33.6 million) (35). The coding of the outcome diagnosis U07.1! described in detail below and the validation of a SARS-CoV-2 infection through a polymerase chain reaction (PCR) test resulted in a subsample of 206 045 individuals. This latter subsample (called “COVID-19 population”) was also used to investigate disease severity among infected persons only (or fatality case in case of death). In addition, we used the larger net-sample (called “total population” in the tables) to investigate overall risks. This approach enabled the study of the risk of hospitalization or mortality for the total population (combined with the risk of having a SARS-CoV-2 infections). It also enabled the study of the risk of hospitalization or death (ie, fatality case) independently of the risks of acquiring an infection for the COVID-19 population (36).

### Observation period

The two-year observation period to measure our outcomes ranged from 1 January 2020 to 31 December 2021, covering the four main infection waves since the beginning of the pandemic in Germany (37).

### COVID-19-outcomes

The two main outcomes were: (i) COVID-19-related hospitalization, defined as a full inpatient hospitalization for which the ICD-10-diagnosis U07.1! was documented on admission as a principal or secondary diagnosis during the observation period; and (ii) COVID-19-related mortality, which included all deaths during the observation period that occurred either (a) within 30 days after a confirmed COVID-19 disease (outpatient COVID-19 disease and treatment), (b) during a COVID-19-related hospitalization or (c) within 14 days of a full inpatient hospitalization with principal or secondary diagnosis U07.1!.

### Pre-existing medical conditions

Pre-existing medical conditions were based on reported ICD-codes in the insurance records and classified into seven categories, following those suggested by the Robert Koch Institute and clustered into seven groups (31): cardiovascular diseases, pulmonary diseases, neurological and psychiatric diseases, liver and kidney diseases, metabolic diseases, cancer, and other pre-existing medical conditions. The coding of the ICD-10 code of the diseases was collected for the total population in the year 2019. For COVID-19 patients with an existing ICD-10 U07.1! code, the last 365 days prior to the index quarter were used to identify a defined coding of pre-existing conditions.

### Occupational groups

Occupations were regrouped into four classifications of the official German national classification scheme, all of which are based on the most recent data available prior to the observation period (2014–2019). This information is available as a 5-digit code that is routinely (on a yearly basis) sent by the employer to the insurance according to the “Classification of Occupations 2010” (KldB 2010) (35, 38). Examples are the code ‘81302’ (health care and nursing). An overview by occupational sector, occupational segment, and main occupational groups is included in the appendix. The first two digits of the KldB can be used to identify 5 broad occupational sectors, which can additionally be divided into 14 smaller occupational segments (as subcategories of sectors). The fourth and fifth digits can be used to ascertain a leadership function (either supervisory or managerial responsibilities) and the fifth digit contains information on the required skill-level of the occupation (four categories). Occupational sectors and occupational segments classify occupations primarily in terms of branch of industry (ie, horizontal dimension) and are the two highest aggregation levels of the KldB 2010. Skill level and leadership function, on the other hand, describe a vertical structure of occupations along different levels of complexity of an occupation or according to the type of leadership function (manager or supervisor). Table 1 presents details of each category and their distribution in the study population.

**Table 1:** Sample description, stratified by type of population. [SD=standard deviation]

Category		Total Population			COVID Population	
		N	% (SD)		N	% (SD)
Age						
	18–67	3 173 171	43.9 (13.1)		206 046	41.9 (12.7)
Sex						
	Female	1 488 462	46.9		101 395	49.2
	Male	1 684 709	53.1		104 651	50.8
Occupational sector (1^st^ & 2^nd^ digit KIdB 2010)						
	Personal Services	611 574	19.7		50 148	24.8
	Business administration and related services	1 096 482	35.4		62 380	30.8
	Other commercial services	357 003	11.5		22 701	11.2
	Service in the IT-sector and the natural sciences	132 205	4.3		6 784	3.4
	Production of goods	900 009	29.1		60 284	29.8
Occupational segment (1^st^ & 2^nd^ digit KldB 2010)						
	Medical and non-medical healthcare	272 775	8.8		24 922	12.3
	Agriculture, forestry and horticulture	28 489	0.9		1358	0.7
	Safety and security occupations	34 048	1.1		1953	1.0
	Cleaning services	59 570	1.9		4 252	2.1
	Business management and organization	460 508	14.9		25 805	12.8
	Business related service occupations	341 759	11.0		18 465	9.1
	Commerce and trade	294 215	9.5		18 110	9.0
	Occupations in traffic and logistics	263 385	8.5		16 496	8.2
	Service in social sector and cultural work	225 014	7.3		18 170	9.0
	Service in the IT-sector and the natural sciences	132 205	4.3		6784	3.4
	Building and interior construction	138 910	4.5		8816	4.4
	Manufacturing	231 637	7.5		17 536	8.7
	Food industry, gastronomy and tourism	113 785	3.7		7056	3.5
	Occupations concerned with production technology	500 964	16.2		32 574	16.1
Skill level (5^th^ digit KldB 2010)						
	Unskilled or semi-skilled	381 362	12.3		27 963	13.8
	Skilled activities	1 912 690	61.8		129 207	63.9
	Complex activities	442 974	14.3		25 864	12.8
	Highly complex activities	360 295	11.6		19 265	9.5
Leadership function ^a^ (4^th^ digit KldB 2010)						
	Manager	77 092	2.4		4840	2.3
	Supervisor	79 392	2.5		4218	2.0
	No	3 016 115	95.1		196 988	95.6
No. of Pre-existing conditions			0.6 (1.02)			0.5 (0.99)
Pre-existing conditions*						
	Cardiovascular diseases	518 219	35.2		29 888	32.8
	Pulmonary diseases	92 272	6.3		5669	6.2
	Neurological and psychiatric diseases	212 982	14.5		13 246	14.6
	Liver and kidney diseases	56 148	3.8		3556	3.9
	Metabolic diseases	167 802	11.4		11 296	12.4
	Cancer	100 314	6.8		5634	6.2
	Other pre-existing conditions	324 117	22.0		21 720	23.9

^a^ In rare cases, due to unusable TTS (eg, no plausible value), no corresponding occupational characteristic could be determined.

### Statistical analysis

After describing the study population (table 1), we presented the number of cases and the cumulative incidence per 100 000 persons for hospitalization and mortality, both for the “total population” (table 2) and “COVID-19-population” (table 3), according to the four occupational classifications. We then presented the results of multivariable regression models for all four occupational characteristics, again for the total (table 4) and COVID-19 (table 5) populations. The results are also shown in figure 1. We estimated a series of Cox proportional hazard regressions with three sets of adjustments. Model 1 investigates associations for each occupational classification separately that are adjusted for age, gender, and region, while model 2 additionally includes the occupational classification of the opposite dimension (eg, skill-level when studying associations for job sector). Model 3 additionally controls for pre-existing medical conditions. The comparison between model 1 and model 2 allowed to investigate, if associations between either vertical (skill level or leadership function) or horizontal (occupation and segment) occupational characteristics persists once we additionally adjusted for the corresponding other dimension of occupation. The comparison between Model 2 and Model 3 can be used to explore whether the associations between occupational characteristics and the COVID-19 outcomes are due to pre-existing medical conditions. The reference category for each exposure is the characteristic with the lowest cumulative incidence. InGef extracted the data tables from the RDB and figures were made with Stata 18.

**Table 2:** COVID-19 hospitalizations and mortality in the **total population** by occupational sector, occupational segment, skill level, leadership function.

Category		N	Hospitalization			Mortality	
			Cases	Cumulative incidence ^a^		Cases	Cumulative incidence ^a^
Occupational sector							
	Personal services	611 574	1571	256.9		72	11.8
	Business administration and related services	1 096 482	2121	193.4		120	10.9
	Other commercial services	357 003	1610	451.0		153	42.9
	Service in the IT sector and natural sciences	132 205	264	199.7		19	14.4
	Production of goods	900 009	3096	344.0		275	30.6
Occupational segment							
	Medical and non-medical healthcare	272 775	744	272.8		26	9.5
	Agriculture, forestry and horticulture	28 489	59	207.0		<5 ^b^	
	Safety and security occupations	34 048	134	393.6		15	44.1
	Cleaning services	59 570	320	537.2		20	33.6
	Business management and organization	460 508	907	197.0		58	12.6
	Business related service occupations	341 759	566	165.6		33	9.7
	Commerce and trade	294 215	648	220.3		29	9.8
	Occupations in traffic and logistics	263 385	1156	438.9		118	44.8
	Service in social sector and cultural work	225 014	483	214.7		25	11.1
	Service in the IT-sector and the natural sciences	132 205	264	199.7		19	14.4
	Building and interior construction	138 910	461	332.0		37	26.6
	Manufacturing	231 637	1000	431.7		106	45.8
	Food industry, gastronomy and tourism	113 785	344	302.3		21	18.5
	Production technology	500 964	1576	314.6		129	25.8
Skill level							
	Unskilled or semi-skilled	381 362	1644	431.1		122	32.0
	Skilled activities	1 912 690	5362	290.3		402	21.0
	Complex activities	442 974	995	224.6		73	16.5
	Highly complex activities	360 295	661	183.5		42	11.7
Leadership function							
	Manager	77 092	189	245.2		20	26.0
	Supervisor	79 392	204	257.0		17	21.4
	No	3 016 115	8777	291.0		669	22.2

^a^ Cumulative incidence per 100 000. ^b^ Due to anonymization regulations, no exact information can be provided.

**Table 3:** COVID-19 hospitalizations and mortality for the **COVID-Population** by occupational sector, occupational segment, skill level and leadership function.

Category		N	Hospitalization			Mortality	
			Cases	Cumulative incidence		Cases	Cumulative incidence ^a^
Occupational sector							
	Personal Services	50 148	1571	3121.7		72	143.6
	Business administration and related services	62 380	2121	3400.1		120	192.4
	Other commercial services	22 701	1610	7092.2		153	674.0
	Service in the IT sector and natural sciences	6784	264	3891.5		19	280.1
	Production of goods	60 284	3096	5135.7		275	456.2
Occupational segment							
	Medical and non-medical healthcare	24 922	744	2985.3		26	104.3
	Agriculture, forestry and horticulture	1358	59	4344.6		<5 ^b^	
	Safety and security occupations	1953	134	6861.2		15	769.1
	Cleaning services	4252	320	7525.9		20	470.4
	Business management and organization	25 805	907	3514.8		58	224.8
	Business related service occupations	18 465	566	3065.3		33	178.7
	Commerce and trade	18 110	648	3578.1		29	160.1
	Occupations in traffic and logistics	16 496	1156	7007.8		118	715.3
	Service in social sector and cultural work	18 170	483	2658.2		25	137.6
	Service in the IT-sector and the natural sciences	6784	264	3891.5		19	280.1
	Building and interior construction	8816	461	5229.1		37	419.7
	Manufacturing	17 536	1000	5702.6		106	604.5
	Food industry, gastronomy and tourism	7056	344	4875.3		21	297.6
	Production technology	32 574	1576	4838.2		129	396.0
Skill level							
	Unskilled or semi-skilled	27 963	1644	5879.2		122	436.3
	Skilled activities	129 207	5362	4149.9		402	311.1
	Complex activities	25 864	995	3847.1		73	282.4
	Highly complex activities	19 265	661	3431.1		42	218.0
Leadership function							
	Manager	4218	189	4480.8		20	474.2
	Supervisor	4840	204	4214.9		17	351.2
	No	196 988	8777	4455.6		669	339.6

^a^ Cumulative incidence per 100 000. ^b^ Due to anonymization regulations, no exact information can be provided.

**Table 4:** Hazard ratio (HR) and 95% confidence interval (CI) for COVID-19 hospitalizations and mortality for the **total population**.

Category		Hospitalization						Mortality				
		Model 1 ^a^		Model 2 ^b^		Model 3 ^c^		Model 1		Model 2		Model 3
		HR (95% CI)		HR (95% CI)		HR (95% CI)		HR (95% CI)		HR (95% CI)		HR (95% CI)
Occupational sector												
	Service in the IT-sector and natural sciences	Ref.		Ref.		Ref.		Ref.		Ref.		Ref.
	Business administration and related services	1.03 (0.91–1.17)		0.99 (0.87–1.13)		1.01 (0.88–1.14)		0.91 (0.56–1.48)		0.87 (0.53–1.42)		0.89 (0.55–1.45)
	Other commercial services	2.04 (1.79–2.32)		1.63 (1.42–1.86)		1.57 (1.37–1.79)		2.35 (1.46–3.79)		1.86 (1.14–3.03)		1.73 (1.06–2.82)
	Production of goods	1.59 (1.40–1.80)		1.41 (1.24–1.60)		1.41 (1.24–1.60)		1.69 (1.06–2.69)		1.47 (0.92–2.35)		1.49 (0.93–2.38)
	Personal Services	1.53 (1.34–1.75)		1.42 (1.24–1.62)		1.41 (1.24–1.61)		1.28 (0.77–2.14)		1.19 (0.71–1.98)		1.18 (0.71–1.97)
Occupational segment ^d^												
	Agriculture, forestry and horticulture	Ref.		Ref.		Ref.		Ref.		Ref.		Ref.
	Service in the IT-sector and the natural sciences	0.90 (0.68–1.19)		1.08 (0.81–1.43)		1.06 (0.80–1.40)		1.27 (0.38–4.31)		1.56 (0.46–5.28)		1.47 (0.43–5.00)
	Business related service occupations	0.81 (0.62–1.06)		0.96 (0.74–1.26)		0.95 (0.73–1.25)		1.06 (0.33–3.48)		1.29 (0.39–4.22)		1.24 (0.38–4.07)
	Business management and organization	0.93 (0.71–1.21)		1.06 (0.82–1.39)		1.06 (0.82–1.39)		1.28 (0.40–4.08)		1.46 (0.46–4.69)		1.41 (0.44–4.53)
	Safety and security occupations	1.52 (1.12–2.07)		1.66 (1.22–2.25)		1.47 (1.08–2.00)		2.79 (0.81–9.63)		3.04 (0.88–10.54)		2.36 (0.68–8.18)
	Commerce and trade	1.08 (0.83–1.41)		1.23 (0.94–1.61)		1.22 (0.93–1.60)		1.07 (0.33–3.53)		1.24 (0.38–4.09)		1.21 (0.37–4.01)
	Food industry. gastronomy and tourism	1.55 (1.18–2.04)		1.56 (1.19–2.06)		1.55 (1.17–2.04)		2.22 (0.66–7.44)		2.23 (0.67–7.50)		2.15 (0.64–7.20)
	Occupations in traffic and logistics	1.76 (1.35–2.28)		1.73 (1.33–2.25)		1.66 (1.27–2.15)		2.98 (0.95–9.39)		2.96 (0.94–9.32)		2.68 (0.85–8.43)
	Building and interior construction	1.32 (1.00–1.73)		1.41 (1.08–1.85)		1.45 (1.10–1.90)		1.74 (0.54–5.64)		1.86 (0.57–6.05)		1.93 (0.59–6.27)
	Occupations concerned with production technology	1.33 (1.02–1.72)		1.49 (1.14–1.93)		1.44 (1.11–1.87)		1.87 (0.59–5.88)		2.10 (0.67–6.61)		1.96 (0.62–6.19)
	Cleaning services	2.40 (1.82–3.18)		2.14 (1.61–2.82)		2.02 (1.53–2.68)		3.20 (0.95–10.78)		2.88 (0.85–9.75)		2.53 (0.75–8.55)
	Manufacturing	1.72 (1.32–2.24)		1.74 (1.34–2.27)		1.71 (1.31–2.22)		3.07 (0.98–9.69)		3.10 (0.98–9.79)		2.96 (0.94–9.34)
	Service in social sector and cultural work	1.13 (0.86–1.48)		1.37 (1.04–1.80)		1.32 (1.01–1.74)		1.45 (0.44–4.80)		1.85 (0.55–6.16)		1.71 (0.51–5.71)
	Medical and non-medical healthcare	1.54 (1.18–2.01)		1.70 (1.30–2.22)		1.65 (1.27–2.16)		1.49 (0.45–4.95)		1.64 (0.49–5.45)		1.55 (0.47–5.13)
Skill level ^e^												
	Highly complex activities	Ref.		Ref.		Ref.		Ref.		Ref.		Ref.
	Complex activities	1.21 (1.09–1.33)		1.20 (1.08–1.32)		1.15 (1.04–1.27)		1.36 (0.93–1.99)		1.33 (0.91–1.96)		1.23 (0.84–1.81)
	Skilled activities	1.61 (1.48–1.74)		1.46 (1.34–1.59)		1.34 (1.23–1.46)		1.94 (1.41–2.66)		1.59 (1.14–2.21)		1.33 (0.96–1.85)
	Unskilled or semi-skilled	2.42 (2.21–2.65)		1.89 (1.71–2.08)		1.67 (1.51–1.84)		2.93 (2.06–4.16)		2.00 (1.37–2.92)		1.57 (1.08–2.30)
Leadership function ^e^												
	Manager	Ref.		Ref.		Ref.		Ref.		Ref.		Ref.
	Supervisor	1.08 (0.89–1.32)		1.00 (0.82–1.22)		0.97 (0.79–1.18)		0.87 (0.46–1.67)		0.73 (0.38–1.40)		0.69 (0.36–1.32)
	No	1.41 (1.22–1.63)		1.21 (1.05–1.40)		1.12 (0.97–1.30)		1.18 (0.76–1.85)		0.91 (0.58–1.43)		0.78 (0.49–1.22)

^a^ Adjusted for age, sex, and region. ^b^ Model1 + occupational characteristics. ^c^ Model 2 + adjusted for pre-existing conditions. ^d^ Model 2 occupational characteristics adjusted for skill level and leadership function. ^e^ Model 2 occupational characteristics adjusted for occupational segment.

**Table 5:** Hazard ratio (HR) and 95% confidence intervals (CI) for COVID-19 hospitalizations and mortality for the **COVID-population**.

Category		Hospitalization						Lethality				
		Model 1 ^a^		Model 2 ^b^		Model 3 ^c^		Model 1		Model 2		Model 3
		HR (95% CI)		HR (95% CI)		HR (95% CI)		HR (95% CI)		HR (95% CI)		HR (95% CI)
Occupational sector												
	Service in the IT-sector and natural sciences	Ref.		Ref.		Ref.		Ref.		Ref.		Ref.
	Business administration and related services	1.03 (0.90–1.17)		1.04 (0.91–1.18)		1.05 (0.92–1.19)		0.91 (0.56–1.48)		0.9 (0.55–1.47)		0.92 (0.56–1.49)
	Other commercial services	1.66 (1.45–1.89)		1.48 (1.29–1.69)		1.43 (1.25–1.63)		1.94 (1.2–3.12)		1.7 (1.05–2.77)		1.59 (0.98–2.58)
	Production of goods	1.22 (1.07–1.38)		1.16 (1.02–1.32)		1.18 (1.04–1.33)		1.32 (0.83–2.1)		1.22 (0.76–1.96)		1.26 (0.79–2.01)
	Personal Services	1.04 (0.91–1.19)		1.01 (0.89–1.16)		1.02 (0.90–1.17)		0.85 (0.51–1.42)		0.82 (0.49–1.38)		0.85 (0.51–1.43)
Occupational segment ^d^												
	Agriculture, forestry and horticulture	Ref.		Ref.		Ref.		Ref.		Ref.		Ref.
	Service in the IT-sector and the natural sciences	0.84 (0.64–1.12)		0.9 (0.68–1.2)		0.89 (0.67–1.18)		1.26 (0.37–4.26)		1.36 (0.4–4.62)		1.32 (0.39–4.47)
	Business related service occupations	0.79 (0.6–1.03)		0.86 (0.66–1.13)		0.86 (0.66–1.12)		1.05 (0.32–3.43)		1.15 (0.35–3.77)		1.14 (0.35–3.75)
	Business management and organization	0.88 (0.67–1.14)		0.95 (0.73–1.24)		0.95 (0.73–1.24)		1.28 (0.4–4.09)		1.37 (0.43–4.4)		1.33 (0.42–4.27)
	Safety and security occupations	1.28 (0.94–1.74)		1.34 (0.99–1.82)		1.22 (0.90–1.66)		2.51 (0.73–8.66)		2.59 (0.75–8.97)		2.1 (0.61–7.27)
	Commerce and trade	0.93 (0.71–1.21)		0.99 (0.76–1.3)		0.98 (0.75–1.28)		1.00 (0.30–3.29)		1.07 (0.32–3.52)		1.04 (0.32–3.44)
	Food industry. gastronomy and tourism	1.24 (0.94–1.64)		1.24 (0.94–1.63)		1.21 (0.92–1.59)		1.82 (0.54–6.11)		1.80 (0.54–6.03)		1.71 (0.51–5.72)
	Occupations in traffic and logistics	1.36 (1.04–1.76)		1.33 (1.02–1.73)		1.28 (0.99–1.67)		2.46 (0.78–7.73)		2.39 (0.76–7.53)		2.21 (0.7–6.94)
	Building and interior construction	0.96 (0.73–1.26)		1.00 (0.76–1.31)		1.03 (0.78–1.35)		1.31 (0.40–4.25)		1.34 (0.41–4.35)		1.44 (0.44–4.67)
	Occupations concerned with production technology	1.00 (0.77–1.29)		1.05 (0.81–1.36)		1.04 (0.80–1.35)		1.52 (0.48–4.77)		1.58 (0.5–4.97)		1.54 (0.49–4.84)
	Cleaning services	1.64 (1.24–2.17)		1.5 (1.13–1.98)		1.41 (1.07–1.87)		2.24 (0.66–7.56)		2.08 (0.62–7.06)		1.8 (0.53–6.11)
	Manufacturing	1.11 (0.86–1.45)		1.11 (0.85–1.45)		1.11 (0.85–1.44)		2.13 (0.68–6.73)		2.09 (0.66–6.6)		2.08 (0.66–6.57)
	Service in social sector and cultural work	0.72 (0.55–0.95)		0.78 (0.59–1.02)		0.78 (0.59–1.02)		0.96 (0.29–3.19)		1.08 (0.33–3.61)		1.08 (0.32–3.59)
	Medical and non-medical healthcare	0.88 (0.67–1.15)		0.92 (0.7–1.2)		0.92 (0.71–1.20)		0.85 (0.26–2.82)		0.87 (0.26–2.9)		0.9 (0.27–2.98)
Skill level ^e^												
	Highly complex activities	Ref.		Ref.		Ref.		Ref.		Ref.		Ref.
	Complex activities	1.1 (1–1.22)		1.07 (0.97–1.18)		1.05 (0.95–1.16)		1.29 (0.88–1.90)		1.24 (0.84–1.83)		1.21 (0.82–1.79)
	Skilled activities	1.31 (1.2–1.42)		1.19 (1.09–1.29)		1.12 (1.03–1.21)		1.68 (1.22–2.32)		1.39 (1.00–1.94)		1.22 (0.87–1.70)
	Unskilled or semi-skilled	1.75 (1.6–1.92)		1.43 (1.29–1.57)		1.31 (1.19–1.44)		2.18 (1.53–3.11)		1.57 (1.07–2.29)		1.31 (0.89–1.91)
Leadership function ^e^												
	Manager	Ref.		Ref.		Ref.		Ref.		Ref.		Ref.
	Supervisor	0.98 (0.8–1.2)		0.95 (0.78–1.15)		0.95 (0.78–1.16)		0.86 (0.45–1.66)		0.75 (0.39–1.45)		0.76 (0.39–1.47)
	No	1.26 (1.09–1.46)		1.17 (1.01–1.36)		1.11 (0.96–1.28)		1.16 (0.73–1.85)		0.96 (0.6–1.53)		0.85 (0.53–1.35)

^a^ Adjusted for age, sex, and region. ^b^ Model1 + occupational characteristics. ^c^ Model 2 + adjusted for pre-existing conditions. ^d^ Model 2 occupational characteristics adjusted for skill level and leadership function. ^e^ Model 2 occupational characteristics adjusted for occupational segment.

**Figure 1a:**
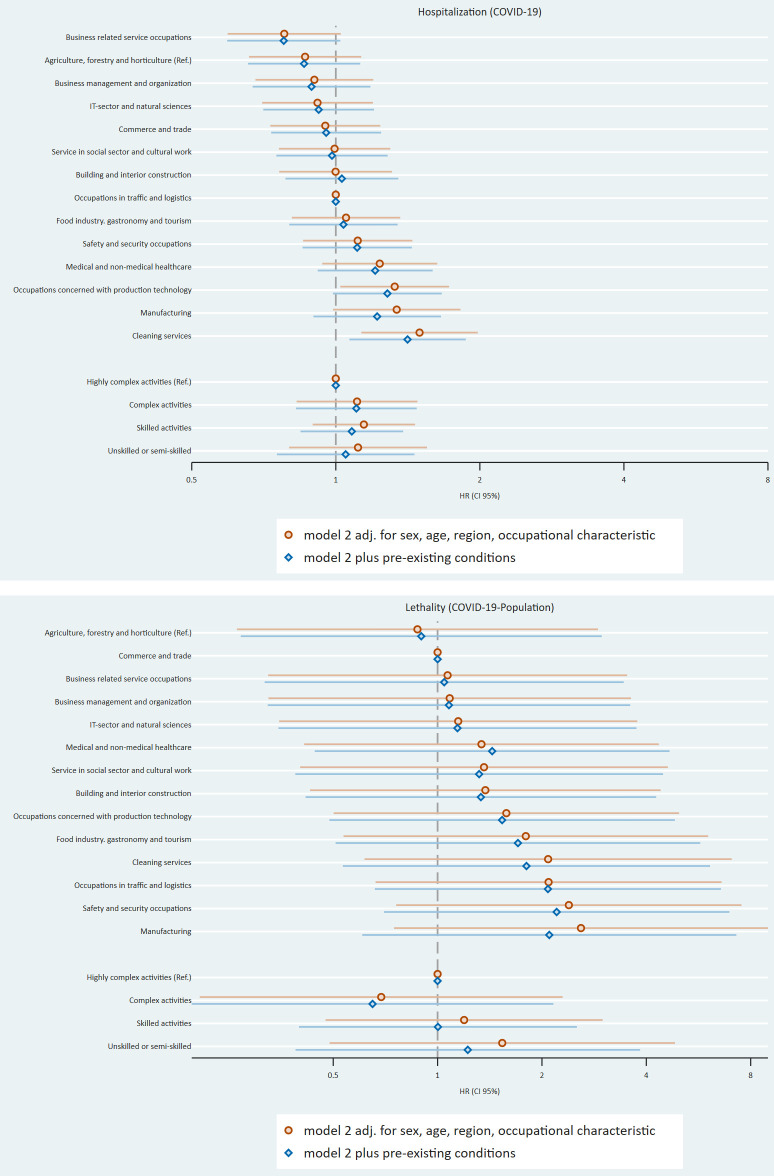
Hazard ratios for COVID-19 outcomes for total population.

**Figure 1b:**
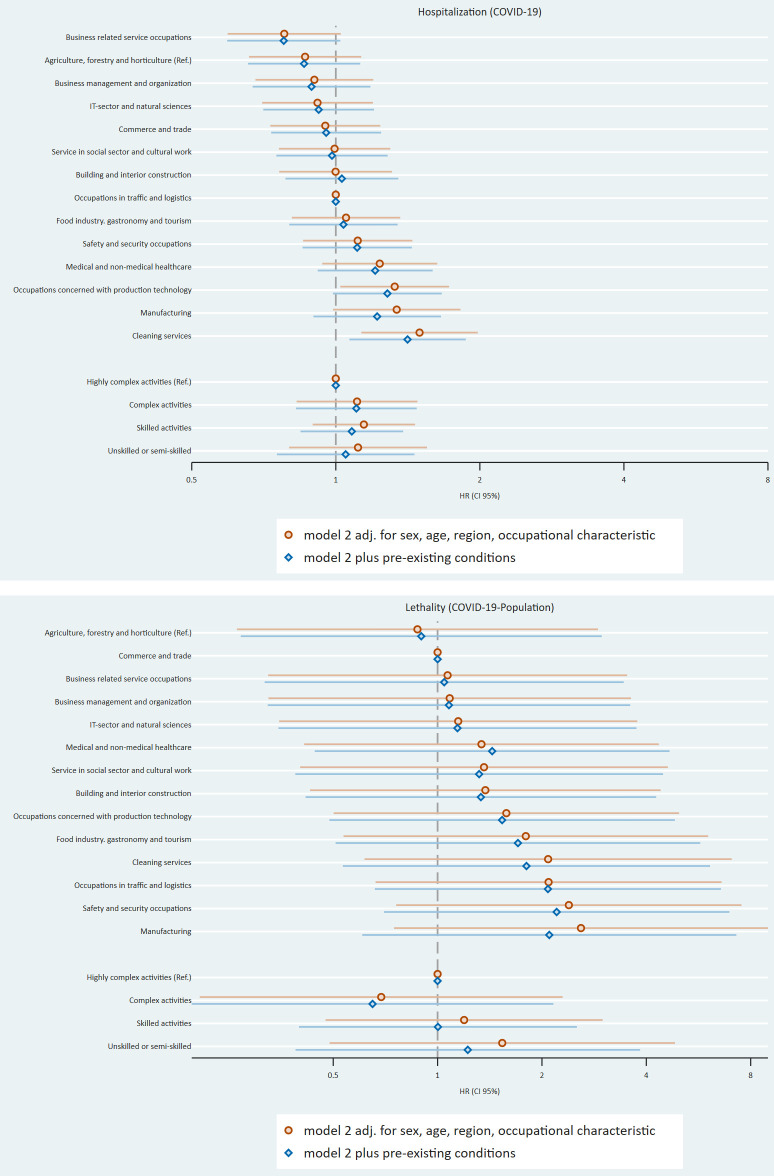
Hazard ratios for COVID-19 outcomes for COVID population.

## Results

### Descriptive results

Table 1 gives an overview of our study sample. Overall, the majority were qualified as professionals or did not hold a leadership function. The sample population most frequently (approximately 16%) worked in the production technology segment. Cardiovascular diseases and neurological and mental diseases were the most common pre-existing medical conditions.

Table 2 presents the number of cases and cumulative incidence for both outcomes (hospitalization and mortality/lethality) across the four occupational characteristics among the total population. Table 3 provides the same information for the COVID-19 population. In both populations and for both outcomes, the occupational segments cleaning services, occupations in traffic and logistics, safety and security services, and manufacturing had the highest cumulative incidences. These were considerably higher in the COVID-19 population. In both populations, individuals in unskilled or semi-skilled positions had the highest cumulative incidence for both outcomes. In the entire population, those without a leadership function had the highest cumulative incidence for hospitalization but the lowest for mortality. In the COVID-19 population, employees with no leadership function and managers had a very similar cumulative incidence for hospitalization. However, the difference between these two for mortality was considerably larger, with a higher mortality among managers.

### Multivariable analysis

Table 4 and 5 present results of our multivariable analyses. Model 1 was used to show the potential association between occupation and COVID-19 outcomes. Compared to the reference category (agriculture, forestry and horticulture), almost all occupational segments have a hazard ratio (HR) of >1 for both outcomes for the total population. For hospitalization, the segments ‘cleaning services’ [HR 2.4, confidence interval (CI) 1.82–3.18], ‘manufacturing’ (HR 1.72, 95% CI 1.32–2.24) and ‘traffic and logistics’ (HR 1.76, 95% CI 1.35–2.58) have the highest HR. Similar results were found for mortality. Looking at the vertical occupational characteristics (skill level and leadership function), all subcategories have an HR >1 for both outcomes compared to the respective reference category. Unskilled or semi-skilled employees have the highest HR for both outcomes (hospitalization: HR 2.42, 95% CI 2.21–2.65; mortality: HR 2.93, 95% CI 2.06–4.16) compared to the other skill levels observed. Employees with no leadership function have a higher HR (hospitalization: HR 1.41, 95% CI 1.22–1.63; mortality: HR 1.18, 95% CI 0.76–1.85) compared to those with a leadership function. Regarding the COVID-19 population, we found an association between occupation and COVID-19 for both outcomes, too, but with smaller HR than for the total population. Here, employees working in the ‘cleaning sector’ have the highest HR (1.64, 95% CI 1.24–2.17) for hospitalization and mortality, too. Employees who are unskilled or semi-skilled and employees with no leadership function have the highest HR in the other occupational categories in comparison to the reference category.

Model 2 (where occupational categories were included simultaneously) shows that the vertical and horizontal job characteristics with the highest HR in the first model remained the same for this model. Looking at the mortality for the general population for the occupational segments, occupations in manufacturing have the highest HR (3.10, 95% CI 0.98–9.79). The HR for the COVID-19-population for hospitalization and lethality are lower than for the total population. Comparing model 2 to model 1, looking at the total population, 11 segments have higher HR and 2 lower HR (cleaning, traffic and logistic) for both outcomes. Looking at the vertical occupational characteristics, all HR are lower comparing model 2 with model 1. Looking at the COVID-19 population, the HR of the horizontal and vertical characteristics for hospitalization behave in the same way as those for the total population. For the lethality, four segments show lower HR, the rest higher HR. The differences between the compared HR were significant only for unskilled- or semiskilled occupations (in both populations).

Model 3 shows that occupations in cleaning services (HR 2.02, 95% CI 1.53–2.86), and manufacturing (HR 1.71, 95% CI 1.31–2.22) have the highest HR for hospitalization in the total population. In this model, manufacturing occupations have the highest mortality, too. The HR of manufacturing occupations do not differ much between the three models. For the COVID-19 population, the influence of pre-existing conditions regarding the association between occupational characteristics and COVID-19 outcomes is observable, too. Comparing the HR of model 3 to those of model 2, 11 segments show lower HR and one higher HR for hospitalization and 12 lower HR for mortality, and all HR lower for the vertical characteristics. For the COVID-19 population, seven HR are lower for the occupational segments for hospitalization and all for the vertical occupational characteristics; For lethality, ten HR are lower for occupational segments, all HR for skill level, and the HR for employees with no managerial responsibly. The differences between the compared HR were not significant.

## Discussion

In this study, we investigated occupational differences of COVID-19 disease severity based on four occupational classifications and using hospitalization and mortality as outcomes. Additionally, our study is the first from Germany that explicitly explores the extent to which differences can be explained by pre-existing medical conditions.

Our main findings can be summarized as follows. First, for each of the four occupational classifications, we can observe risk differences in hospitalization and in mortality. Regarding horizontal classifications, we determined that people working in the commercial service sector (especially cleaning or traffic and logistics) or the production sector (especially manufacturing) have higher risks for either of the two outcomes. Turning to hierarchical classifications, we found a clear gradient in the case of skill level (with higher risk for lower skill levels for both outcomes) and some support for higher hospitalization among workers without leadership function (but no differences for mortality). Importantly, these differences persisted in multivariable analyses that not only adjusted for age, sex, and region, but also for the corresponding other dimension of occupation – thus pointing to independent effects of the two dimensions of occupations (vertical and horizontal). Furthermore, differences were observed for the total sample and also when we restricted the sample to those who had a SARS-CoV-2 infection (COVID-population).

Second, with regard to a potential explanation via pre-existing health conditions, we observed that associations between occupation and the two COVID-19 outcomes were generally weakened once we introduced health condition into the multivariable models. Most notably, this is the case for occupational skill level. This weakened associations points to a possible partial mediation, indicating that people who worked in unskilled occupations may have been more likely to be in poor health, which in turn could explain their higher risk of severe COVID-19 disease. Again, we found this pattern for both the total and COVID-19 populations.

Our first finding contributes to the existing knowledge. As we investigated four complementary occupational classifications in relation to two different outcomes (based on a large sample and multivariable analyses that adjust for potential confounders), we add to existing knowledge that uses often only horizontal occupations as variables. In addition, by mutually adjusting for the occupational classifications as part of the multivariable analyses, we were able to detect independent effects, showing that previously documented associations (eg, for segments) are not simply due to other characteristics of these occupations (eg, lower skill levels in specific segments).

Our second finding (pointing to potential mediation), is also in line with previous research, specifically studies pointing to higher risk of disease severity for people with poorer health (39). Yet, as our study also includes information on occupation, we were able to investigate the complex interrelations between occupation, pre-existing conditions, and COVID-19 severity in more detail, and thereby, found some support that occupational differences are partly explained by poorer health conditions in low-skilled occupations.

This leads to another insight of our research, specifically that pre-existing conditions seem to be more important for differences by skill-levels, while their explanatory role is smaller for segments and sectors. The reason for this could also be that health differences are also more pronounced by skill levels than segments because workers in lower-skilled occupations are subject to a disproportionately higher risk of adverse health outcomes (40) due to a combination of occupational, socioeconomic and healthcare-related factors Low-skilled workers, for example, face higher health risks due to occupational hazards, including physically demanding tasks, exposure to harmful substances, and limited job autonomy (41–43). Chronic stress, poor working circumstances, and socioeconomic disadvantages contribute to higher rates of conditions such as cardiovascular disease and diabetes, which elevate the risk of severe illness (42, 43). This combination of physical and psychosocial strain, and pre-existing health issues leads to long-term health deterioration and poorer health conditions, that in turn increase the risk of disease severity (41).

### Strengths and limitations

Our study has several strengths. We used administrative health insurance data, which is less prone to reporting and selection bias compared to self-reported survey data. Core outcomes such as infection and hospitalization are based on routinely collected claims data, minimizing misclassification. In addition, participants were selected according to predefined inclusion criteria (see the Methods section), reducing the risk of selection effects typically associated with differential response rates in survey data. This is particularly important for socioeconomically disadvantaged groups, who are often underrepresented in survey-based studies. In addition, the large sample size available in the InGef database enhances statistical power, allows for detailed analyses across occupational groups, and makes it possible to focus specifically on infected individuals when examining disease severity.

The administrative data used in this study, however, also entail key limitations (44) as key variables were unavailable and could not be considered in our analyses. This includes, for example, information on infection control measures within jobs and the inability to account for additional potential confounders. While multivariable analyses ruled out the possibility that the observed associations were driven by gender, age, or region of employment, other important factors—such as nationality or commuting patterns (eg, use of public transportation)—could not be included. Additional variables that may influence infection risk but were not included are individual risk behaviors, health literacy, and distinctions between full- and part-time employment. Limitations also apply to the measurement of our COVID-19 outcomes. Although case identification was based on laboratory-confirmed diagnoses reported to health insurers, it cannot be ruled out that certain occupational groups may be underrepresented in these data due to differences in health behavior (eg, delayed symptom recognition or later healthcare-seeking behavior) (45, 46). In this context, some workers may have been less likely of testing and to obtain a confirmatory laboratory test following a positive self-test. A German study (47) estimates that up to 45% of COVID-19 infections remained undetected, with this share being slightly higher in socioeconomically disadvantaged regions. It is therefore possible that the infection risk for certain occupational groups (eg, cleaning staff, transport and logistics workers, or agricultural workers) may be underestimated. Yet, as robust empirical analyses on this issue are lacking and because we focus on disease severity (and not on infection risk only), it remains unclear how this may have affected the results presented here. Likewise, data on COVID-19 vaccination status were not available in our data, as vaccinations in Germany were primarily recorded outside the statutory health insurance system (eg, in personal vaccination booklets). As a result, we could not adjust for differences in vaccination uptake.

Another important limitation relates to data protection regulations, which are particularly strict in the context of administrative health data. These restrictions limited our ability to conduct more refined mediation analyses—for instance, by testing indirect effects or applying advanced causal mediation analyses (48) —and confined us to simpler comparisons between models with and without the proposed mediator (49). As a result, our ability to formally assess mediation pathways remains constrained. These techniques would also have allowed for a more detailed investigation of the role of pre-existing conditions, including potential confounding and moderation effects—an important aspect raised in the introduction that could not be addressed in the present study, or would have required additional efforts that go beyond the initial aims of the project. Another limitation concerns the adjustment for comorbidities. Pre-existing health conditions were identified based on pre-defined diagnoses, grouped into broader disease groups. While this approach allows for an overall assessment of health status, it does not distinguish between long-standing and newly diagnosed conditions - although both reflect the general health profile of individuals in their respective occupational group. Furthermore, by aggregating different conditions, we were unable to isolate the specific contribution of individual diseases to the association between occupation and COVID-19 outcomes.

### Concluding remarks

Our study is the first in Germany that used a large database to demonstrate that occupational differences in COVID-19 disease severity – particularly in vertical occupational characteristics – can in part be explained by pre-existing medical conditions. Our study supports the hypothesis that the pandemic increases existing health inequalities, so that already disadvantaged population groups are further disadvantaged by an accumulation of risk factors. Addressing occupational health disparities in non-pandemic times may therefore increase health equity in a future pandemic.

## Supplementary material

Supplementary material
