# Supplementary material for: Can pre-existing medical conditions explain occupational differences in COVID-19 disease severity? An analysis of 3.17 million people insured in Germany
Source: Scand J Work Environ Health. 2025 Aug 29;51(5):380–93. doi: 10.5271/sjweh.4242 (PMC12412139; doi:10.5271/sjweh.4242)
Supplement: Supplementary material [file SJWEH-51-380-S001.pdf]

Can pre-existing medical conditions explain occupational differences in COVID-19 disease severity? An analysis of 3.17 million people insured in Germany[1]

by Robert Guţu, MSc,[2] Valerie Schaps, MSc, Benjamin Wachtler, MD, Florian Beese, MSc, Jens Hoebel, PhD, Marco Alibone, MSc, Morten Wahrendorf, PD, PhD

1. Supplementary material
2. Correspondence to: Robert Guțu, Institute of Medical Sociology, Centre for Health and Society, Medical Faculty and University Hospital Düsseldorf, Heinrich-Heine University Düsseldorf, Moorenstraße 5, 40225 Düsseldorf, Germany. [E-mail: r.gutu@hhu.de]

Table S1 Affiliation of Occupational Groups to Occupational Segments and Occupational Sectors.

Occupational sector	Occupational segment	Main occupational groups
Production of goods	Agriculture, forestry and horticulture	Agricultural, Animal Care, and Forestry Occupations
		Horticultural and Floristry Professions
	Building and interior construction	Building Planning, Architecture, and Surveying Professions
		Civil Engineering Professions (Building and Structural Construction)
		Interior and Building Finishing Professions
		Building and Supply Engineering Professions
	Occupations concerned with production technology	Mechanical and Vehicle Engineering Professions
		Mechatronik-, Energie- und Elektroberufe
		Technical Research, Development, Design, and Production Control Professions
	Manufacturing	Raw Material Extraction and Processing, Glass and Ceramics Manufacturing and Processing
		Plastics Manufacturing and Processing, Woodworking and Wood Processing
		Paper and Printing Professions, Technical Media Design
		Metal Production and Processing, Metal Construction Professions
		Textile and Leather Professions
		Product Design and Craft Professions, Fine Arts, Musical Instrument Making
Personal Services	Food industry. gastronomy and tourism	Food Production and Processing Professions
		Tourism, Hotel, and Restaurant Professions
	Service in social sector and cultural work	Education, Social and Domestic Professions, Theology
		Teaching and Training Professions

		Language, Literature, Humanities, Social and Economic Sciences Professions
		Performing and Entertaining Professions
	Medical and non-medical healthcare	Medical and Health Professions
		Non-Medical Health, Personal Care, and Wellness Professions, Medical Technology
Business administration and related services	Commerce and trade	Purchasing, Sales, and Trade Professions
		Sales Professions
	Business related service occupations	Professions in Financial Services, Accounting, and Tax Consulting
		Professions in Law and Administration
		Advertising, Marketing, Commercial, and Editorial Media Professions
	Business management and organization	Professions in Business Management and Organization
Service in the IT-sector and the natural sciences	Service in the IT-sector and the natural sciences	Mathematics, Biology, Chemistry, and Physics Professions
		Geology, Geography, and Environmental Protection Professions
		Information Technology (IT), Information, and Communication Technology Professions
Other commercial services	Safety and security occupations	Protection, Security, and Surveillance Professions
		Members of the Regular Armed Forces
	Occupations in traffic and logistics	Transport and Logistics Professions (excluding Vehicle Operation)
		Operators of Vehicles and Transport Equipment
	Cleaning services	Cleaning Professions

1. Beese F, Waldhauer J, Wollgast L, Pförtner T-K, Wahrendorf M, Haller S, et al. Temporal dynamics of socioeconomic inequalities in COVID-19 outcomes over the course of the pandemic—a scoping review. International journal of public health. 2022;67:1605128.

2. Bartig S, Beese F, Wachtler B, Grabka MM, Mercuri E, Schmid L, et al. Socioeconomic differences in SARS-CoV-2 infection and vaccination in Germany: a seroepidemiological study after one year of COVID-19 vaccination campaign. International journal of public health. 2023;68:1606152.

3. Olivella-Cirici M, Perez G, Rodríguez-Sanz M, Forcadell-Díez L, Cejas PM, Pasarín MI. Socioeconomic inequalities in the incidence of COVID-19 in Barcelona students. Public Health in Practice. 2024;8:100527.

4. Wang L, Swayze S, Bodner K, Calzavara A, Harrigan SP, Siddiqi A, et al. Social inequalities in COVID-19 deaths by area-level income: patterns over time and the mediating role of vaccination in a population of 11.2 million people in Ontario, Canada. medRxiv. 2024:2024.01. 15.24301331.

5. Billingsley S, Brandén M, Aradhya S, Drefahl S, Andersson G, Mussino E. COVID-19 mortality across occupations and secondary risks for elderly individuals in the household: a population register-based study. Scandinavian journal of work, environment & health. 2021;48(1):52.

6. Bonde JPE, Begtrup LM, Jensen JH, Flachs EM, Schlünssen V, Kolstad HA, et al. Occupational risk of SARS-CoV-2 infection: a nationwide register-based study of the Danish workforce during the COVID-19 pandemic, 2020–2021. Occupational and Environmental Medicine. 2023;80(4):202-8.

7. Bonde JPE, Sell L, Flachs EM, Coggon D, Albin M, Oude Hengel KM, et al. Occupational risk of COVID-19 related hospital admission in Denmark 2020–2021: a follow-up study. Scandinavian journal of work, environment & health. 2022;49(1):84.

8. Guțu R, Schaps V, Hoebel J, Wachtler B, Beese F, Jacob J, et al. Berufsbedingte Unterschiede in COVID-19-Erkrankungen – Eine wellenspezifische Analyse von 3,17 Millionen gesetzlich Versicherten. 2023.

9. Nwaru CA, Santosa A, Franzén S, Nyberg F. Occupation and COVID-19 diagnosis, hospitalisation and ICU admission among foreign-born and Swedish-born employees: a register-based study. J Epidemiol Community Health. 2022;76(5):440-7.

10. Wahrendorf M, Schaps V, Reuter M, Hoebel J, Wachtler B, Jacob J, et al. Berufsbedingte Unterschiede bei COVID-19-Morbidität und-Mortalität in Deutschland. Eine Analyse von Krankenkassendaten von 3, 17 Mio. Versicherten. Bundesgesundheitsblatt-Gesundheitsforschung-Gesundheitsschutz. 2023;66(8):857-68.

11. Biarnés-Martínez M, Fàbregas M, Coma E, Pera G, Fina F, Rivera-Arco A, et al. SARS-CoV-2 infection in occupational settings in Catalonia. Revista Espanola de Salud Publica. 2022;96:e202205040-e.

12. Romero Starke K, Mauer R, Hegewald J, Bolm-Audorff U, Brückner G, Schüssel K, et al. Occupational risks of COVID-19: a case-cohort study using health insurance claims data in Germany. BMC Public Health. 2024;24(1):3235.

13. Ballering AV, Oertelt-Prigione S, Initiative LCR, olde Hartman TC, Rosmalen JG. Sex and gender-related differences in COVID-19 diagnoses and SARS-CoV-2 testing practices during the first wave of the pandemic: The Dutch lifelines COVID-19 cohort study. Journal of Women's Health. 2021;30(12):1686-92.

14. Beale S, Hoskins S, Byrne T, Fong WLE, Fragaszy E, Geismar C, et al. Differential risk of SARS-CoV-2 infection by occupation: evidence from the Virus Watch prospective cohort study in England and Wales. Journal of Occupational Medicine and Toxicology. 2023;18(1):5.

15. Nafilyan V, Pawelek P, Ayoubkhani D, Rhodes S, Pembrey L, Matz M, et al. Occupation and COVID-19 mortality in England: a national linked data study of 14.3 million adults. Occupational and Environmental Medicine. 2022;79(7):433-41.

16. Mutambudzi M, Niedzwiedz C, Macdonald EB, Leyland A, Mair F, Anderson J, et al. Occupation and risk of severe COVID-19: prospective cohort study of 120 075 UK Biobank participants. Occupational and environmental medicine. 2021;78(5):307-14.

17. Verbeeck J, Vandersmissen G, Peeters J, Klamer S, Hancart S, Lernout T, et al. Confirmed COVID-19 cases per economic activity during autumn wave in Belgium. International Journal of Environmental Research and Public Health. 2021;18(23):12489.

18. Nakamura T, Mori H, Saunders T, Chishaki H, Nose Y. Impact of workplace on the risk of severe COVID-19. Frontiers in Public Health. 2022;9:731239.

19. Torén K, Albin M, Bergström T, Murgia N, Alderling M, Schiöler L, et al. Occupational risks associated with severe COVID-19 disease and SARS-CoV-2 infection–a Swedish national case-control study conducted from October 2020 to December 2021. Scandinavian Journal of Work, Environment & Health. 2023;49(6):386.

20. Rhodes S, Beale S, Daniels S, Gittins M, Mueller W, McElvenny D, et al. Occupation and SARS-CoV-2 in Europe: a review. European Respiratory Review. 2024;33(173).

21. Diderichsen F, Evans T, Whitehead M. The social basis of disparities in health. Challenging inequities in health: From ethics to action. 2001;1:12-23.

22. Farmer P. Social inequalities and emerging infectious diseases. Understanding and applying medical anthropology. 2016:118-26.

23. Quinn SC, Kumar S. Health Inequalities and Infectious Disease Epidemics: A Challenge for Global Health Security. Biosecurity and Bioterrorism: Biodefense Strategy, Practice, and Science. 2014;12(5):263-73.

24. Waldhauer J, Beese F, Wachtler B, Haller S, Koschollek C, Pförtner T-K, et al. Socioeconomic differences in the reduction of face-to-face contacts in the first wave of the COVID-19 pandemic in Germany. BMC Public Health. 2022;22(1):2419.

25. Wahrendorf M, Reuter M, Hoebel J, Wachtler B, Höhmann A, Dragano N. Regional disparities in SARS-CoV-2 infections by labour market indicators: a spatial panel analysis using nationwide German data on notified infections. BMC Infectious Diseases. 2022;22(1):661.

26. Wachtler B, Beese F, Demirer I, Haller S, Pförtner T-K, Wahrendorf M, et al. Education and pandemic SARS-CoV-2 infections in the German working population–the mediating role of working from home. Scandinavian Journal of Work, Environment & Health. 2024;50(3):168.

27. Bartig S, Beese F, Wachtler B, Grabka MM, Mercuri E, Schmid L, et al. Socioeconomic Differences in SARS-CoV-2 Infection and Vaccination in Germany: A Seroepidemiological Study After One Year of COVID-19 Vaccination Campaign. International Journal of Public Health. 2023;Volume 68 - 2023.

28. Bartley M, Kelly-Irving M. Health inequality: an introduction to concepts, theories and methods: John Wiley & Sons; 2024.

29. AlAlawneh K-N, Ibrahim R, Mhaidat Q, Suliman M, Rababah A, Ayasreh H, et al. Effect of Pre-Existing Chronic Medical Conditions on Clinical Features and Disease Outcomes of COVID-19 Infection in Jordanian Children. Journal of Biosciences and Medicines. 2023;11(12):275-86.

30. Ebinger JE, Achamallah N, Ji H, Claggett BL, Sun N, Botting P, et al. Pre-existing traits associated with Covid-19 illness severity. PloS one. 2020;15(7):e0236240.

31. Marušić J, Hasković E, Mujezinović A, Đido V. Correlation of pre-existing comorbidities with disease severity in individuals infected with SARS-COV-2 virus. BMC Public Health. 2024;24(1):1053.

32. Rößler M, Jacob J, Risch L, Tesch F, Enders D, Wende D, et al. Hierarchisierung von Risikofaktoren für schwere COVID-19-Erkrankungsverläufe im Kontext der COVID-19-Schutzimpfungen. 2021.

33. Treskova-Schwarzbach M, Haas L, Reda S, Pilic A, Borodova A, Karimi K, et al. Pre-existing health conditions and severe COVID-19 outcomes: an umbrella review approach and meta-analysis of global evidence. BMC medicine. 2021;19:1-26.

34. Ludwig M, Enders D, Basedow F, Walker J, Jacob J. Sampling strategy, characteristics and representativeness of the InGef research database. Public health. 2022;206:57-62.

35. Hoffmann W, Latza U, Baumeister SE, Brünger M, Buttmann-Schweiger N, Hardt J, et al. Guidelines and recommendations for ensuring Good Epidemiological Practice (GEP): a guideline developed by the German Society for Epidemiology. European journal of epidemiology. 2019;34:301-17.

36. für Arbeit B. Klassifikation der Berufe 2010–überarbeitete Fassung 2020 Band 1: Systematischer und alphabetischer Teil mit Erläuterungen. Nürnberg URL: https://statistik arbeitsagentur de/DE/Statischer-Content/Grundlagen/Klassifikationen/Klassifikation-der-Berufe/KldB2010-Fassung2020/Printausgabe-KldB-2010-Fassung2020/Generische-Publikationen/KldB2010-PDF-Version-Band1-Fassung2020 pdf. 2021.

37. Westreich D, Edwards JK, Tennant PW, Murray EJ, Van Smeden M. Choice of outcome in COVID-19 studies and implications for policy: mortality and fatality. American Journal of Epidemiology. 2022;191(2):282-6.

38. Tolksdorf K, Loenenbach A, Buda S. Dritte Aktualisierung der „Retrospektiven Phaseneinteilung der COVID-19-Pandemie in Deutschland “. 2022.

39. Matthes B, Meinken H, Neuhauser P. Berufssektoren und Berufssegmente auf Grundlage der KldB 2010. Methodenbericht der Statistik der BA, Nürnberg. 2015;8.

40. Burgard SA, Lin KY. Bad jobs, bad health? How work and working conditions contribute to health disparities. American Behavioral Scientist. 2013;57(8):1105-27.

41. Cui J, Ren Y-H, Zhao F-J, Chen Y, Huang Y-F, Yang L, et al. Cross-sectional study of the effects of job burnout on immune function in 105 female oncology nurses at a tertiary oncology hospital. Medical Science Monitor: International Medical Journal of Experimental and Clinical Research. 2021;27:e929711-1.

42. Karasek Jr RA. Job demands, job decision latitude, and mental strain: Implications for job redesign. Administrative science quarterly. 1979:285-308.

43. Theorell T. Healthy work: Stress, productivity and the reconstruction of working life: Basic Books.; 1992.

44. Höhne J, Buschoff KS. Die Arbeitsmarktintegration von Migranten und Migrantinnen in Deutschland. Ein Überblick nach Herkunftsländern und Generationen. WSI-Mitteilungen. 2015;68(5):345-54.

45. Islamoska S, Petersen JH, Benfield T, Norredam M. Socioeconomic and demographic risk factors in COVID-19 hospitalization among immigrants and ethnic minorities. European Journal of Public Health. 2022;32(2):302-10.

46. Klein J, von dem Knesebeck O. Soziale Unterschiede in der ambulanten und stationären Versorgung. Bundesgesundheitsblatt-Gesundheitsforschung-Gesundheitsschutz. 2016;59(2):238-44.

47. Biggerstaff M, Cauchemez S, Reed C, Gambhir M, Finelli L. Estimates of the reproduction number for seasonal, pandemic, and zoonotic influenza: a systematic review of the literature. BMC infectious diseases. 2014;14(1):1-20.

48. Neuhauser H, Buttmann-Schweiger N, Ellert U, Fiebig J, Hövener C, Offergeld R, et al. Seroepidemiologische Studien zu SARS-CoV-2 in Stichproben der Allgemeinbevölkerung und bei Blutspenderinnen und Blutspendern in Deutschland–Ergebnisse bis August 2021. 2021.

49. VanderWeele T. Explanation in causal inference: methods for mediation and interaction: Oxford University Press; 2015.
